# The role of primary lymph node sites in survival and mortality prediction in Hodgkin lymphoma: a SEER population‐based retrospective study

**DOI:** 10.1002/cam4.1280

**Published:** 2018-03-09

**Authors:** Amr Ebied, Vuong Thanh Huan, Omar Mohamed Makram, To Kim Sang, Mohamed Ghorab, Huyen Thi Ngo, Ahmed Iraqi, Mohamed Gomaa Kamel, Tran Ngoc Dang, Nguyen Lam Vuong, Kenji Hirayama, Nguyen Tien Huy

**Affiliations:** ^1^ Egyptian National Blood Transfusion Services Cairo Egypt; ^2^ Online Research Club (http://www.onlineresearchclub.org/) Nagasaki Japan; ^3^ Pham Ngoc Thach University of Medicine Ho Chi Minh City Vietnam; ^4^ Faculty of Medicine October 6 University Giza 12566 Egypt; ^5^ Ho Chi Minh City Oncology Hospital Ho Chi Minh City Vietnam; ^6^ Faculty of Medicine Alexandria University Alexandria Egypt; ^7^ University of Medicine and Pharmacy at Ho Chi Minh City Ho Chi Minh City Vietnam; ^8^ Cairo University Hospitals Giza Egypt; ^9^ Faculty of Medicine Minia University Minia Egypt; ^10^ Department of Immunogenetics Institute of Tropical Medicine (NEKKEN) Graduate School of Biomedical Sciences Nagasaki University 1‐12‐4 Sakamoto Nagasaki 852‐8523 Japan; ^11^ Evidence Based Medicine Research Group & Faculty of Applied Sciences Ton Duc Thang University Ho Chi Minh City Vietnam; ^12^ Department of Clinical Product Development Institute of Tropical Medicine (NEKKEN) Leading Graduate School Program, and Graduate School of Biomedical Sciences Nagasaki University 1‐12‐4 Sakamoto Nagasaki 852‐8523 Japan

**Keywords:** Epidemiology, Hodgkin lymphoma, primary lymph node site, public health, survival

## Abstract

As diagnostic and therapeutic modalities for Hodgkin's Lymphoma (HL) continue to improve, patient‐related factors affecting survival become more difficult to identify. Very little is known about the relationship between the primary site of lymph node (LN) involvement and survival of HL patients. We retrospectively analyzed the United States Surveillance, Epidemiology and End Results (SEER) database for 12,658 HL patients reported between 1973 and 2010 using survival analysis and time‐interval multiple logistic regression (MLR) to disclose that relationship. The effect of all primary LN sites on the survival of HL patients was supported. The intra‐abdominal (IAB) primary LN site was significantly associated with the worst survival. The pelvic (P) LN sites were significantly and independently associated with nearly 2 times and 2.5 times the probability of having 1‐year overall mortality (OM) and 1‐year cancer‐specific mortality (CSM), respectively. Head, face and neck (HFN) primary LN sites were significant and independent predictors of better overall and HL‐specific survival. A worse survival with the intra‐abdominal primary LN site was probably related to their association with higher age, or advanced stages of HL. The biological basis behind the aggressiveness of intra‐abdominal and pelvic LN sites is yet to be investigated.

## Introduction

Hodgkin lymphoma (HL) is a lymphoid malignancy arising from B‐lymphocytes of the lymph nodes’ (LN) germinal centers. In the United States (US), the estimated HL cases and deaths in 2010 were 8490 and 1320, respectively 1. It is estimated that 8260 new HL cases and 1070 new deaths will occur in the US in 2017 2. Morphologically, and in accordance with the 2008 World Health Organization (WHO) classification, HL was categorized into two main subtypes; classical (cHL) and nodular lymphocyte predominant Hodgkin lymphoma (NLPHL). The former can be further subdivided into four distinct entities; lymphocyte‐rich (LR), lymphocyte‐depleted (LD), mixed cellularity (MC), and nodular sclerosis (NS) 3.

With the subsequent advancement in diagnostic techniques used in determining the spread of each of the above entities of HL, came the need to include classification systems for HL's clinical course and the extent to guide therapeutic decisions. For example, the Ann Arbor classification took disease spread into account, and later, its modification; the Cotswold staging system, provided more variables related to both prognosis and extranodal extension 4. The Lugano classification was later introduced to highlight the higher sensitivity of Positron Emission Tomography (PET) scans in the early assessment of extranodal involvement 5.

With the introduction of more treatment modalities to HL, more focus has been directed to prognostic factors that would affect the choice of chemotherapeutic agents and predict the survival of HL patients after treatment 6, 7. However, as treatment modalities for HL continued to improve, factors associated with patients’ survival grew more difficult to identify. Originally, an international prognostic score was developed for advanced HL to determine patients who were more likely to benefit from conventional treatment versus those who would need more sophisticated lines of treatment. This score was made up of seven unfavorable parameters, including a serum albumin < 4 g/dL, a hemoglobin level < 10.5 g/dL, a patient's age ≥ 45 years, male gender, Ann Arbor stage IV, leukocytosis ≥ 15,000 cells/mm^3^, and lymphopenia < 600 lymphocytes/mm^3^ 8. Prognostic criteria for HL have been extended in recent years to include patients with early disease, as well as those who had been shown to include the presence of a mass in the mediastinum, an elevated sedimentation rate, multiple LN sites, age > 50 years, and splenomegaly or enlargement of any other extranodal sites 9, 10. Yet, the idea that the primary site of LN involvement can have an impact on the survival of patients with HL has never been raised in sufficient detail in the literature before. In this retrospective cohort study, we aimed to disclose that impact.

## Methods

### Data

Data from 1973 to 2010 were retrospectively obtained from the United States Surveillance, Epidemiology and End Results (SEER) database, which comprises about 28% of the US population 11. Authorization for access to the SEER database was granted after registration with the SEER web site and without emphasis on specific ethical or review board approvals. HL cases were defined in the SEER database according to the third edition of the International Classification of Diseases for Oncology (ICD‐O‐3) coding system and in compliance with the InterLymph Consortium classification of lymphoid neoplasms for epidemiological research based on the 2008 WHO classification 12, 13. Case listings having HL were requested using the National Cancer Institute SEER*stat 8.3.4 software. Within the same case listing session, a number of factors were requested as column variables for the desired table output, including each patient's age, year of diagnosis, gender, race, lymphoma subtype, radiotherapy status, surgery status, sequence number of HL among other cancers, marital status at diagnosis, Ann Arbor stage, vital status at the end of follow‐up, SEER cause‐specific death classification, mean survival months, and our predictor of interest, the primary site of LNs involved. The primary outcomes of this analysis were overall survival (OS), cancer‐specific survival (CSS), 1‐, 5‐, and 10‐year overall (OM) and cancer‐specific mortality (CSM).

### Patient selection

**Figure 1:**
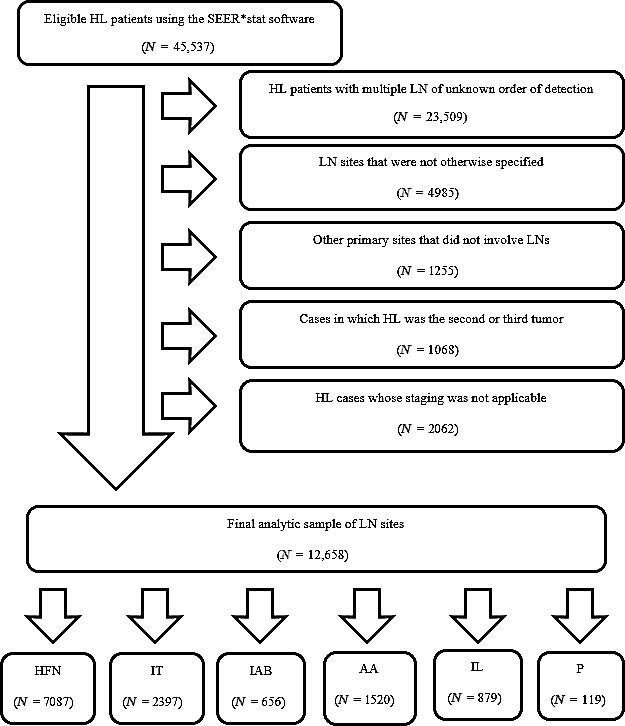
The methodology of patient selection using the SEER*stat software. LN, Lymph nodes; HL, Hodgkin lymphoma; HFN, Head, face and neck; IT, Intrathoracic; IAB, Intra‐abdominal; AA, Axilla and arm; IL, Inguinal region and leg; P, Pelvic.

Figure**** 1 illustrates the methodology used to select patients for the final analytic sample. Subsequently, primary LN sites were categorized into six specific LN node groups defined by the SEER database.

### Coding of data

For Ann Arbor staging purposes, the years of diagnosis were categorized into four main intervals (1973–1982, 1983–1987, 1988–2003, and 2004–2010). Table S1 (not shown) shows how these intervals were used, together with disease extension codes; EOD 4‐extent, EOD 10‐extent, and clinical stage (CS) to accurately define individual stages of the HL analytic sample. Exclusion of HL cases whose staging was not applicable led to the exclusion of HL cases that were diagnosed between 1973 and 1982.

### Statistical analysis

Table outputs of the above‐mentioned data were then analyzed using JMP 10 software (SAS Institute, Cary, NC). Summary statistics, including means and standard deviations, were provided for continuous predictors, such as age and year of diagnosis. Frequencies and proportions were used to summarize discrete variables. Chi‐square and correlation tests were used to compare proportions of respective discrete and continuous epidemiological, clinical, and pathological characteristics across the selected six categories of primary LN sites. Survival curves were generated using the Kaplan–Meier (KM) method, and the log‐rank tests were performed to compare survival differences. Hazard ratios (HR) along with their 95% confidence intervals (CI) were calculated using univariable and multivariable Cox proportional hazards regression (PHR) models to determine how different variable levels were associated relatively, as well as individually, with survival. MLR models for 1‐, 5‐, and 10‐year OM, as well as CSM, were generated, and the Odds ratios (OR) of the probabilities of associated mortalities were calculated for each of the selected primary LN sites to provide an additional evidence for the association between primary LN sites and mortality.

For the OM MLR models, patients with the outcome (0 =  dead) were selected by first categorizing the survival months into ≤12, ≤60, and ≤120 months, and then only keeping the patients who are dead in that period as dead, while keeping all the rest as alive. Accordingly, the vital status based on that selection was subsequently recoded into (0 =  dead) and (1 =  alive). For the CSM MLR, deaths that were solely attributed or caused by HL diagnosis, and have already occurred within 1, 5, and 10 years from diagnosis were recoded as (0 =  dead), whereas other patients who were either alive or dead due to another cause at any time within the study time frame were recoded as (1 =  alive). The analytic sample (*n* = 12,658) was divided into training (70% = 8861), and validation (30% = 3797) sets. For each time interval (1‐, 5‐, and 10‐year), MLR model was performed using the training data. Receiver operating characteristic (ROC) curve with the area under the curve (AUC) were recorded, and confusion matrices for the training and validation data were used to calculate the misclassification ratios and the accuracy of the training and validation data of each model. For our suggested regression models, the head, face and neck primary LN site was chosen to be the reference group, because it had the largest number of observations and was the LN site with consistently significant results. For all the above statistical tests, a *P*‐value of less than 0.05 was considered statistically significant.

## Results

### Patients’ characteristics

Table 1 shows patient characteristics across all selected primary LN sites. There were significant differences in age (in years) and survival time (in months) across the primary LN sites, with the highest values belonging to the intra‐abdominal site (approximate mean age of 58 years, and mean survival time of 63 months, respectively), followed by the primary pelvic site (approximate mean age of 55 years, and mean survival time of 60 months, respectively) (*P*‐value < 0.0001). With respect to gender, there were significantly more males than females across all selected primary LN sites, with the exception of the intrathoracic LNs, where females constituted almost 53% of all 2397 HL patients with primary intrathoracic LNs (*P*‐value < 0.0001). The proportion of White HL patients across all primary LN sites was significantly the largest (*P*‐value < 0.0001).

**Table 1:** Basic characteristics of the sample cohort (N = 12,658) across all selected primary LN sites[N(%)]

	Total	HFN	IT	IAB	AA	IL	P	*P*‐value
Characteristics	(*N* = 12,658)	(*N* = 7087)	(*N* = 2397)	(*N* = 656)	(*N* = 1520)	(*N* = 879)	(*N* = 119)	
Survival (in months)								
Mean ± SD^c^	99.24 ± 83.77	106.37 ± 85.01	101.62 ± 84.22	63.37 ± 73.87	85.81 ± 77.6	90.65 ± 79.9	59.87 ± 68	<0.0001
Patient age (years)								
Mean ± SD^c^	40.7 ± 19.53	37.89 ± 19.24	36.29 ± 15.9	58.29 ± 18.18	47.73 ± 19.11	48.78 ± 19.18	55.48 ± 18.88	<0.0001
Year of diagnosis								
1983–1991	2328 (18.39)	1369 (19.32)	408 (17.02)	135 (20.58)	252 (16.58)	153 (17.41)	11 (9.24)	0.0002
1992–2001	4191 (33.11)	2358 (33.27)	797 (33.25)	200 (30.49)	500 (32.89)	307 (34.93)	29 (24.37)	
2002–2010	6139 (48.5)	3360 (47.41)	1192 (49.73)	321 (48.93)	768 (50.53)	419 (47.67)	79 (66.39)	
Patient gender								
Male	7140 (56.41)	4014 (56.64)	1137 (47.43)	399 (60.82)	897 (59.01)	628 (71.44)	65 (54.62)	<0.0001
Female	5518 (43.59)	3073 (43.36)	1260 (52.57)	257 (39.18)	623 (40.99)	251 (28.56)	54 (45.38)	
Race								
White	10,673 (84.32)	6020 (84.94)	2073 (86.48)	559 (85.21)	1204 (79.21)	717 (81.57)	100 (84.03)	<0.0001
Black	1381 (10.91)	700 (9.88)	214 (8.93)	60 (9.15)	258 (16.97)	134 (15.24)	15 (12.61)	
Other^a^	494 (3.9)	289 (4.08)	101 (4.21)	36 (5.49)	47 (3.09)	18 (2.05)	3 (2.52)	
Unknown	110 (0.87)	78 (1.10)	9 (0.38)	1 (0.15)	11 (0.72)	10 (1.14)	1 (0.84)	
Lymphoma Subtype								
LR	697 (5.51)	421 (5.94)	20 (0.83)	27 (4.12)	131 (8.62)	88 (10.01)	10 (8.4)	<0.0001
MC	2252 (17.79)	1438 (20.29)	149 (6.22)	152 (23.17)	301 (19.80)	189 (21.50)	23 (19.33)	
LD	174 (1.37)	62 (0.87)	27 (1.13)	42 (6.4)	24 (1.58)	16 (1.82)	3 (2.52)	
NS	6910 (54.59)	3934 (55.51)	1772 (73.93)	207 (31.55)	630 (41.45)	331 (37.66)	36 (30.25)	
cHL, NOS	1865 (14.73)	876 (12.36)	421 (17.56)	193 (29.42)	206 (13.55)	129 (14.68)	40 (33.61)	
NLPHL	760 (6.00)	356 (5.02)	8 (0.33)	35 (5.34)	228 (15.00)	126 (14.33)	7 (5.88)	
Radiation therapy								
Yes	5702 (45.05)	3369 (47.54)	1221 (50.94)	61 (9.30)	674 (44.34)	350 (39.82)	27 (22.69)	<0.0001
No	6956 (54.95)	3718 (52.46)	1176 (49.06)	595 (90.70)	846 (55.66)	529 (60.18)	92 (77.31)	
Surgery								
Yes	2469 (19.51)	1552 (21.90)	352 (14.69)	23 (3.51)	341 (22.43)	187 (21.27)	14 (11.76)	<0.0001
No	10,189 (80.49)	5535 (78.10)	2045 (85.31)	633 (96.49)	1179 (77.57)	692 (78.73)	105 (88.24)	
Sequence number								
One primary only	11,511 (90.94)	6466 (91.24)	2234 (93.20)	578 (88.11)	1341 (88.22)	779 (88.62)	113 (94.96)	<0.0001
First of two or more	1147 (9.06)	621 (8.76)	163 (6.80)	78 (11.89)	179 (11.78)	100 (11.38)	6 (5.04)	
Marital status at diagnosis								
Married	5921 (46.78)	3064 (43.23)	1169 (48.77)	372 (56.71)	791 (52.04)	457 (51.99)	68 (57.14)	<0.0001
Not married^b^	6737 (53.22)	4023 (56.77)	1228 (51.23)	284 (43.29)	729 (47.96)	422 (48.01)	51 (42.86)	
Stage								
I	6921 (54.68)	4044 (57.06)	1114 (46.47)	210 (32.01)	949 (62.43)	552 (62.80)	52 (43.7)	<0.0001
II	3150 (24.89)	1747 (24.65)	827 (34.50)	182 (27.74)	235 (15.46)	127 (14.45)	32 (26.89)	
III	745 (5.89)	410 (5.79)	110 (4.59)	54 (8.23)	104 (6.84)	51 (5.80)	16 (13.45)	
IV	1038 (8.2)	379 (5.35)	258 (10.76)	180 (27.44)	129 (8.49)	73 (8.30)	19 (15.97)	
Unknown	804 (6.35)	507 (7.15)	88 (3.67)	30 (4.57)	103 (6.78)	76 (8.65)	0 (0.00)	

SD, Standard deviation; NLPHL, Nodular Lymphocytic Predominant Hodgkin Lymphoma; LR, Lymphocyte‐rich; LD, Lymphocyte‐depleted; MC, Mixed cellularity; NS, Nodular sclerosis; cHL, classical Hodgkin Lymphoma, LN, Lymph Nodes, HFN, Head, face, and neck; IT, Intrathoracic; IAB, Intra‐abdominal; AA, Axilla or arm; IL, Inguinal region or leg; P, Pelvic.

a Other= Includes American Indian, AK Native, Asian, and Pacific Islander.

b Not married= Includes single, widowed, separated, and divorced.

c Mean ± SD was calculated using student's t‐test for continuous variables that were normally distributed.

Interestingly, regarding the distribution of primary LN sites with respect to HL staging, it was found that the intra‐abdominal primary LN sites were associated with more advanced stages of HL. Although primary LN sites of head, face and neck, intrathoracic, axilla or arm, inguinal region or leg, and pelvic regions occurred at percentages of about 11, 15, 15, and 14, and 29, respectively, during stages III and IV of HL, the intra‐abdominal primary LN site occurred in about 36% of advanced cases of HL (*P*‐value < 0.0001). It was equally interesting to notice that only 0.33% of primary intrathoracic LN sites occurred with NLPHL (*P*‐value < 0.0001).

### Survival analysis

**Figure 2:**
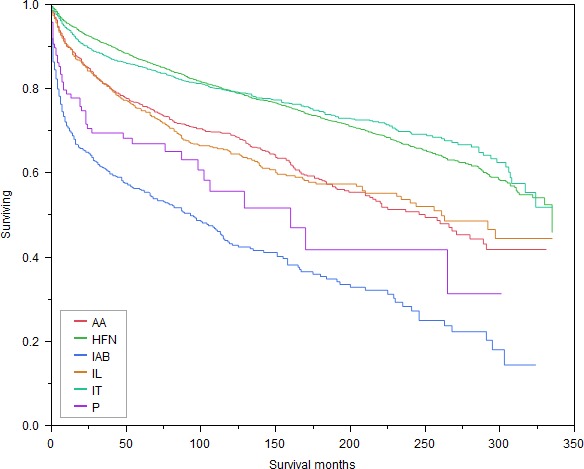
Kaplan–Meier survival curves for OS based on all primary LN sites. HFN, Head, face and neck; IT, Intrathoracic; IAB, Intra‐abdominal; AA, Axilla and arm; IL, Inguinal region and leg; P, Pelvic; OS, Overall survival.

**Figure 3:**
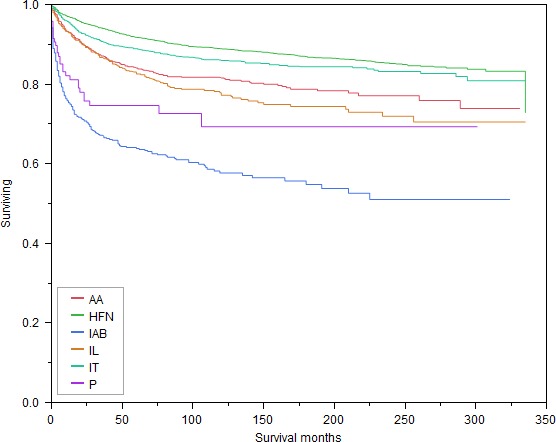
Kaplan–Meier survival curves for CSS based on all primary LN sites. HFN, Head, face and neck; IT, Intrathoracic; IAB, Intra‐abdominal; AA, Axilla and arm; IL, Inguinal region and leg; P, Pelvic; CSS, Cancer‐specific survival.

Patients with primary disease of the intra‐abdominal LN sites had the worst OS (Log‐Rank *P*‐value = < 0.0001, Fig. 2), as well as the worst CSS (Log‐Rank *P*‐value = <0.0001, Fig. 3) among all six LN sites. Figures S2 and S3 (not shown) showed that the OS and CSS of patients with HL varied significantly with all independent variables including the primary site of LNs of the disease, with the exception of gender (Log‐Rank *P*‐value = 0.4267), race (Log‐Rank *P*‐value = 0.0662), and marital status (Log‐Rank *P*‐value = 0.5457) which were found to show no significant differences in CSS.

### Cox proportional hazards regression models

Using the univariable Cox PHR model to predict OS in Table**** 2, it can be stated that increasing age was associated with worsening survival. Compared to NLPHL, all cHL subtypes predicted worse survival, with the greatest hazard of mortality noted for the LD cHL subtype (HR = 5.84, 95% CI = 4.37–7.81, *P*‐value < 0.0001). The risk of mortality among married patients was 10% more than among their nonmarried peers (HR = 1.1, 95% CI = 1.03–1.18, *P*‐value = 0.0061). The first of two or more primary tumors (HR = 1.64, 95% CI = 1.49–1.79, *P*‐value < 0.0001) was found to be a predictor of worse survival. Stages I and II of HL predicted better survival (Stage I HR = 0.81, 95% CI = 0.7–0.94, *P*‐value = 0.0073 and Stage II HR = 0.77, 95% CI = 0.66–0.91, *P*‐value = 0.0018), while stage IV (HR = 1.98, 95% CI = 1.68–2.34, *P*‐value < 0.0001) predicted worse survival. With respect to the head, face and neck primary LN groups as a reference, all other LN sites, with the exception of intrathoracic LNs, were significant predictors of worse survival, with the most risk exhibited by the primary intra‐abdominal LN sites (HR = 3.9, 95% CI = 3.47–4.39, *P*‐value < 0.0001). Using the multivariable Cox PHR model to predict the adjusted association between OS and different variables, it can be shown that patients’ age and year of diagnosis demonstrated similar trends to the univariable model. As regards our predictor of interest, we found that all primary LN sites, excluding pelvic LN sites, to be significant independent predictors of worse OS.

**Table 2:** Univariable and Multivariable Cox proportional hazards regression models for predictors of OS and CSS in the analytic cohort

Characteristics	Overall survival			Overall survival			Cancer‐specific survival			Cancer‐specific survival		
	Univariable Cox PHR model			Multivariable Cox PHR model			Univariable Cox PHR model			Multivariable Cox PHR model		
	HR	P‐value	95% CI	HR	P‐value	95% CI	HR	P‐value	95% CI	HR	P‐value	95% CI
Patient age (years)												
Risk change per year of age	1.06	<0.0001	1.058–1.062	1.06	<0.0001	1.059–1.063	1.05	<0.0001	1.049–1.055	1.05	<0.0001	1.048–1.054
Year of diagnosis												
Risk change per year	0.98	<0.0001	0.978–0.989	0.97	<0.0001	0.965–0.976	0.98	<0.0001	0.976–0.989	0.97	<0.0001	0.962–0.976
Patient gender												
Male	1	Reference		1	Reference		1	Reference		1	Reference	
Female	0.85	<0.0001	0.79–0.91	0.68	<0.0001	0.63–0.74	0.96	0.4270	0.87–1.06	0.79	<0.0001	0.72–0.88
Race												
White	1	Reference		1	Reference		1	Reference		1	Reference	
Black	1.005	0.9354	0.89–1.13	1.25	0.0004	1.1–1.4	1.02	0.7934	0.87–1.19	1.2	0.0276	1.02–1.41
Other^a^	1.13	0.1944	0.94–1.36	1.09	0.3728	0.9–1.3	1.08	0.5442	0.83–1.38	1.1	0.5614	0.83–1.38
Unknown	0.24	<0.0001	0.08–0.51	0.3	0.001	0.11–0.66	0.25	0.002	0.06–0.65	0.29	0.0087	0.07–0.77
Lymphoma subtype												
NLPHL	1	Reference		1	Reference		1	Reference		1	Reference	
LR	1.76	<0.0001	1.37–2.29	1.4	0.0110	1.08–1.82	2.02	0.0012	1.31–3.17	1.61	0.0308	1.04–2.53
MC	2.9	<0.0001	2.34–3.63	1.52	0.0001	1.22–1.9	4.7	<0.0001	3.3–6.95	2.4	<0.0001	1.68–3.56
LD	5.84	<0.0001	4.37–7.81	1.7	0.0005	1.26–2.3	12.6	<0.0001	8.28–19.65	3.41	<0.0001	2.21–5.37
NS	1.5	0.0001	1.21–1.87	1.44	0.0006	1.17–1.81	2.62	< 0.0001	1.85–3.85	2.33	<0.0001	1.64–3.44
cHL, NOS	2.89	<0.0001	2.32–3.64	1.78	<0.0001	1.42–2.25	5.06	<0.0001	3.54–7.51	2.8	<0.0001	1.95–4.18
Radiation therapy												
Radiotherapy	1	Reference		1	Reference		1	Reference		1	Reference	
No Radiotherapy	1.79	<0.0001	1.67–1.93	1.37	<0.0001	1.26–1.5	2.33	<0.0001	2.1–2.59	1.64	<0.0001	1.44–1.88
Surgery												
Surgery	1	Reference	1	Reference	1	Reference	1	Reference				
No Surgery	1.66	< 0.0001	1.49–1.84	1.09	0.1857	0.96–1.23	2.08	<0.0001	1.79–2.43	1.06	0.5608	0.88–1.28
Sequence number												
One primary only	1	Reference		1	Reference		1	Reference		1	Reference	
First of two or more primaries	1.64	<0.0001	1.49–1.79	0.98	0.7187	0.89–1.08	1.18	0.0259	1.02–1.37	0.75	<0.0001	0.65–0.87
Marital status at diagnosis												
Not married^b^	1	Reference		1	Reference		1	Reference		1	Reference	
Married	1.1	0.0061	1.03–1.18	0.67	<0.0001	0.62–0.72	0.97	0.5462	0.88–1.07	0.65	<0.0001	0.59–0.72
Stage												
Unknown	1	Reference		1	Reference		1	Reference		1	Reference	
I	0.81	0.0073	0.7–0.94	0.95	0.5292	0.82–1.11	0.71	0.001	0.58–0.86	0.87	0.1987	0.72–1.07
II	0.77	0.0018	0.66–0.91	1.08	0.3353	0.92–1.28	0.77	0.0185	0.62–0.96	1.08	0.5098	0.87–1.34
III	1.14	0.2021	0.93–1.38	1.2	0.0668	0.98–1.46	1.25	0.0847	0.97–1.62	1.25	0.0902	0.97–1.61
IV	1.98	<0.0001	1.68–2.34	1.68	<0.0001	1.41–1.99	2.51	<0.0001	2.03–3.12	1.96	<0.0001	1.58–2.45
Primary LN site												
LNs of HFN	1	Reference		1	Reference		1	Reference		1	Reference	
LNs of IAB	3.91	<0.0001	3.47–4.39	1.18	0.0102	1.04–1.34	5.2	<0.0001	4.48–6.02	1.55	<0.0001	1.32–1.82
IL LNs	1.82	<0.0001	1.6–2.06	1.18	0.0132	1.04–1.34	2.15	<0.0001	1.81–2.53	1.42	0.0001	1.19–1.68
IT LNs	1.01	0.8606	0.91–1.12	1.22	0.0003	1.1–1.36	1.29	0.0004	1.12–1.47	1.39	<0.0001	1.21–1.6
P LNs	2.78	<0.0001	2.03–3.71	1.2	0.2555	0.87–1.6	3.48	<0.0001	2.35–4.95	1.45	0.0670	0.97–2.07
LNs of AA	1.75	<0.0001	1.58–1.94	1.17	0.0049	1.05–1.3	1.85	<0.0001	1.59–2.13	1.28	0.0012	1.1–1.49

NLPHL, Nodular Lymphocytic Predominant Hodgkin Lymphoma; LR, Lymphocyte‐rich; LD, Lymphocyte‐depleted; MC, Mixed cellularity; NS, Nodular sclerosis; cHL, classical Hodgkin Lymphoma; LN, Lymph Nodes; HFN, Head, face, and neck; IT, Intrathoracic; IAB, Intra‐abdominal; AA, Axilla or arm; IL, Inguinal region or leg; P, Pelvic; OS, Overall survival; CSS, Cancer‐specific survival.

a Other = Includes American Indian, AK Native, Asian, and Pacific Islander,

b Not married = Includes single, widowed, separated, and divorced.

Applying the univariable Cox PHR model for CSS, it can be shown that the same trends of positive and negative predictors of survival continued with respect to mortalities specifically attributed to the diagnosis of HL. The intra‐abdominal primary LN groups continued to be the most significant predictor of worse survival (HR = 5.2, 95% CI = 4.48–6.02, *P*‐value < 0.0001). Gender, race, and marital status were not significant predictors of CSS using the univariable model. Applying the multivariable Cox PHR model, the same trends in the previous multivariable OS model continued, with a single difference, a significant independent prediction of better CSS survival by the first HL of two or more primaries. All primary LN sites, with the exception of the P site, were still significant independent predictors of worse CSS.

### Time‐interval multiple logistic regression

Using the data obtained from Table**** 3 into our extended analysis revealed another interesting observation. The primary pelvic LN site was significantly associated with a 2 times and 2.5 times of the probability of having OM and CSM for only the 1‐year models, thereby exceeding the OR for intra‐abdominal LN sites for the same time intervals. Table**** 4 shows accuracy measures are for all six MLR models (1‐, 5‐, and 10‐year OM, and 1‐, 5‐, and 10‐year CSM). Comparing the accuracy of the six MLR models, it was found that the 1‐year CSM model provided the best accuracy measures (AUC‐ROC = 0.89801, accuracy [training set] = 0.95023, accuracy [validation set] = 0.95365).

**Table 3:** Odds ratios (OR)s and P‐values for 1‐, 5‐, and 10‐year Overall Mortality (OM) as well as Cancer‐Specific Mortality (CSM) for the different primary LN sites selected for the analytic sample using Multiple Logistic Regression (MLR)

Multiple Logistic Regression Model (Training set)	1‐year OM		5‐year OM		10‐year OM		1‐year CSM		5‐year CSM		10‐year CSM	
Primary LN site	OR	P‐value	OR	P‐value	OR	P‐value	OR	P‐value	OR	P‐value	OR	P‐value
LNs of HFN	1	Ref.	1	Ref.	1	Ref.	1	Ref.	1	Ref.	1	Ref.
LNs of IAB	1.97	<0.0001	1.45	0.0048	1.25	0.0808	1.99	<0.0001	1.75	<0.0001	1.56	0.0008
IL LNs	1.4	0.0504	1.47	0.0023	1.53	0.0003	1.42	0.0785	1.64	0.0005	1.73	<0.0001
IT LNs	1.54	0.0029	1.62	<0.0001	1.57	<0.0001	1.47	0.0204	1.69	<0.0001	1.66	<0.0001
P LNs	2.06	0.0285	1.35	0.2972	1.03	0.9125	2.48	0.0108	1.84	0.0447	1.55	0.1387
LN of AA	1.45	0.0093	1.39	0.0021	1.16	0.1443	1.37	0.0604	1.45	0.002	1.26	0.0432

LN, Lymph Nodes; HFN, Head, face, and neck; IT, Intrathoracic; IAB, Intra‐abdominal; AA, Axilla or arm; IL, Inguinal region or leg; P, Pelvic.

**Table 4:** Comparison of six multiple logistic regression models

Multiple Logistic Regression	Training		Validation
	AUC ROC	Accuracy	Accuracy
1‐year OM	0.89589	0.93398	0.93310
5‐year OM	0.84693	0.87044	0.87253
10‐year OM	0.85449	0.85352	0.85199
1‐year CSM	0.89801	0.95023	0.95365
5‐year CSM	0.82718	0.89673	0.90545
10‐year CSM	0.81962	0.88251	0.88675

AUC, Area under the curve; ROC, Receiver operating characteristic; OM, Overall mortality; CSM, Cancer‐specific mortality.

## Discussion

To the best of our knowledge, the impact of specific primary LN sites on the survival and prognosis of HL patients has not been previously mentioned in the medical literature. A number of prior studies described either more generalized primary LN sites of involvement within the context of their survival analyses for HL or were reluctant to draw significant association between primary LN sites and survival in HL 8, 14, 15, 16, 17. Hasenclever and his colleagues, in a cohort of 4677 HL patients derived from 25 centers, stated that inguinal involvement was significantly associated with a 5‐year OS of 73%; yet, he supplemented his univariate survival analysis with the notion that since the sample size was large, most of the factors included were expected to be significant 8. Other studies referred to the site of LN involvement in HL as “supradiaphragmatic” and “infradiaphragmatic” or “subdiaphragmatic” 14, 15, 16, 17. Vassilakopoulos and his colleagues compared the outcomes of 54 patients with clinical stage I/II infradiaphragmatic HL treated with ABVD or equivalent regimens with or without radiotherapy, to those of 444 patients with pure supradiaphragmatic disease, who were treated at the same center, and found that the former group's 10‐year OS was 74 ± 8% (vs. 91 ± 2%, *P*‐value = 0.0006) 14. Glimelius and his colleagues analyzed treatment outcomes of 99 Swedish patients (86 with supradiaphragmatic and 13 with infradiaphragmatic HL) and found that for patients with infradiaphragmatic disease, the OS, HL‐specific survival at 5 and 10 years were all 85%, whereas for patients with supradiaphragmatic disease, the 5‐ and 10‐year OS were 77 and 61%, and the 5‐ and 10‐year HL‐specific survival were 81% and 70%, respectively 15. Darabi et al. retrospectively compared two groups of 1013 patients treated from 1988 to 1993 in two prospective randomized clinical trials in Germany for early and intermediate stages of HL, and found worse outcomes with infradiaphragmatic HL as compared to the supradiaphragmatic variety which he related to its association with known adverse prognostic risk factors, but infradiaphragmatic HL per se, was not an independent adverse prognostic factor for treatment failure or survival 16. Hull and his coworkers also assessed long‐term outcomes of 21 patients with infradiaphragmatic HL, and showed that its 10‐year OS was 70% 17. Referring to our Table S2 describing 1‐, 5‐, and 10‐year survival, and given the aforementioned OS and CSS data provided by the above studies, we can state that our 5‐ and 10‐year OS and CSS rates fell too extreme with respect to previous studies. In comparison to the results of Vassilakopoulos and Hull, we documented lower 10‐year OS and CSS rates for both infra‐ and supradiaphragmatic HL. As for Glimelius's study, our results for the 5‐year OS and CSS were lower for infradiaphragmatic HL, but higher for supradiaphragmatic HL, an observation most probably attributed to differences in sample sizes. It is also worth mentioning that Glimelius's investigation, which included only 99 patients, was the only study that showed that infradiaphragmatic HL had better 5‐year survival outcomes.

We reported a mean age of 40.7 years for the whole analytic sample. In our study, age was a consistent and significant predictor of worse survival using all our statistical methods. Our mean age at diagnosis did not fall far from that provided by previous studies investigating prognostic factors for HL in the US and abroad 6, 18, 19, 20. The results of our study suggest the increased occurrence of cases having intra‐abdominal primary LN sites in advanced stages of HL when compared to other selected primary LN sites. Such an observation was not supported by previously published data. Olu‐Eddo and his colleagues, in their analysis of clinical and pathologic features of 56 HL patients in Nigeria over a study period of 25 years, found that the cervical group of LNs occurred in 78.5% of HL patients identified by biopsy and that 66.1% of those patients had advanced stages of the disease 21.

We also demonstrated that almost no primary intrathoracic LNs occurred with NLPHL. More effort should be put to investigate such an observation, and to correlate findings with both survival and mortality in patients with such a malignant tumor of low prevalence. NLPHL is a disease marked by an indolent course, good therapeutic response, and spontaneous remission. Curable relapses are frequent, but NLPHL can progress to non‐Hodgkin lymphoma (NHL) more frequently than cHL 22, 23. Further investigations into this suggested clinical clue to determine if the presence or absence of primary intrathoracic LNs can influence progression or transformation of NLPHL into NHL will be appreciated.

Applying time‐interval‐based logistic regression into our survival analysis introduced two very interesting observations. First, the 1‐year CSM model showed the highest accuracy indicators. Second, the primary occurrence of HL in the pelvic LN sites was significantly associated with the greatest probability of 1‐year OM and CSM, followed by its occurrence in intra‐abdominal LN sites. These two observations shed new light on the hidden aggressiveness of HL occurring primarily in pelvic LN sites. This may provide additional evidence to previously published data describing the poorer survival of HL occurring primarily in infradiaphragmatic LNs. We also recommend using a similar method of survival analysis for determining the effect of independent variables of very small group sizes within very large population cohorts.

Although the 2016 revision of the WHO classification of lymphoid neoplasms did not provide a big difference for HL from the pathological point of view, other than what has been stated in the 2008 classification, yet, more efforts are needed to analyze newly discovered clinical and pathological observations affecting survival, extrapolate adding significant survival factors to existing prognostic models, and ultimately search for a biological explanation for unusual aggressive behaviors in HL 24, 25. In our intra‐abdominal primary LN sample, advanced age could be the cause for the worse survival of HL, and LD lymphoma subtype could explain poor HL survival. It has also been shown that MC subtype was associated with worse prognosis 26. A biological explanation should be sought for the aggressiveness of the intra‐abdominal primary LN groups at hand. The rising belief of a clonal similarity between the different components of complex lymphomas; such as follicular lymphoma (FL) and mucosa‐associated lymphoid tissue (MALT) lymphoma, or HL and FL or mantle cell lymphoma (MCL) paves the way to simplify the complex appearance of these tumors, which may share other more subtle but common tumor behaviors 25. It has been demonstrated that low‐grade FL could be transformed into highly aggressive diffuse large B‐cell lymphoma (DLBCL), and that histologic transformation could be clinically associated with the abrupt explosive growth of a single LN site, leading to a median survival time of 6–20 months 27, 28. The resulting transformed lymphoma in the previous example has the same lineage; yet, other types of lymphomas, collectively known as composite lymphomas were described to have different lineages (B and T‐cells) or compromising HL as well as NHL combinations 29. Common biomarkers within the cells of aggressive lymphomas, or within cells of composite lymphomas, may explain the aggressive behaviors of primary or transformed lymphomas, or the occurrence of composite lymphomas of different degrees of aggressiveness.

We took a path that was different from that taken by previous studies, and that was to confirm our survival analysis results with time‐interval MLR models; yet, our study still had some limitations. First, the SEER database did not provide other clinical, laboratory, and chemotherapeutic data that we could also have used to derive more accurate predictive models for HL mortality. Second, we were not able to include B‐symptoms within our suggested model, because extracting B‐symptoms using the SEER database led to the acquisition of too many missing data, the exclusion of which would have affected the reality of our sample. Third, the relatively small group sizes of the intra‐abdominal and pelvic LN sites prevented us from extending our analysis to the pediatric population, or doing the same survival analysis separately for different levels of the included independent variables to test if there was an effect modification by any of the included patients’ characteristics. Future prospective or retrospective studies, using equal proportions of primary LN sites and incorporating more clinical and treatment‐related variables, are warranted to help researchers compare the presented odds ratios of mortality for the included primary LN sites at different levels of the included independent variables (males vs. females, married vs. unmarried, etc.).

In view of the demonstrated impact of primary LN sites on the survival and mortality prediction in HL patients, different clinicopathologic characteristics of patients with primary HL in head, face and neck, intra‐abdominal, or pelvic LN sites should be sought to see if similarities existed that would indicate either resemblance or transformation to a more aggressive or indolent tumor type. We further encourage more studies to be started to compare survival of HL patients affecting the aforementioned LN sites with other unfavorable variables in recent international prognostic scores, with the recommendation of including intra‐abdominal and/or pelvic LN sites as a novel unfavorable prognostic factor for HL‐specific mortality if they continued to stand out as predictors of worse survival in further published work.

## Conflicts of Interest

The authors declare no potential conflicts of interest.

## Supporting information


**Table S1.** Ann Arbor staging criteria based on EOD* 10 ‐ extent (1988–2003) CS** extension (2004+); EOD 4 ‐ extent (1983–1987) according to the SEER database.
**Table S2.** Time‐interval overall survival (OS) and cancer‐specific survival (CSS) across all patient characteristics using the Kaplan–Meier (KM) curves
**Figure S2.** Kaplan–Meier Curves for OS based on all included basic patient characteristics
**Figure S3.** Kaplan–Meier Curves for CSS based on all included basic patient characteristics.
