# Supplementary material for: The role of primary lymph node sites in survival and mortality prediction in Hodgkin lymphoma: a SEER population‐based retrospective study
Source: Cancer Med. 2018 Mar 9;7(4):953–65. doi: 10.1002/cam4.1280 (PMC5911631; doi:10.1002/cam4.1280)
Supplement: Supplementary file 1 — Table S1. Ann Arbor staging criteria based on EOD* 10 ‐ extent (1988–2003) CS** extension (2004+); EOD 4 ‐ extent (1983–1987) according to the SEER database. Table S2. Time‐interval overall survival (OS) and cancer‐specific survival (CSS) across all patient characteristics using the Kaplan–Meier (KM) curves Figure S2. Kaplan–Meier Curves for OS based on all included basic patient characteristics Figure S3. Kaplan–Meier Curves for CSS based on all included basic patient characteristics. [file CAM4-7-953-s001.pdf]

The role of primary lymph node sites in survival and mortality prediction in Hodgkin Lymphoma: A SEER population-based retrospective study.

Supplementary materials for online publication

Supplementary table 1: Ann Arbor staging criteria based on EOD* 10 - extent (1988-2003); CS extension (2004+); EOD 4 - extent (1983-1987) according to the SEER database:**

**Stage I**
1983-1987: EOD 4 - extent (1983-1987) = 1
1988-2003: EOD 10 - extent (1988-2003) = 10-11
2004+: CS extension (2004+) = 100-120
**Stage II**
1983-1987: EOD 4 - extent (1983-1987) = 2
1988-2003: EOD 10 - extent (1988-2003) = 20-21
2004+: CS extension (2004+) = 200-230
**Stage III**
1983-1987: EOD 4 - extent (1983-1987) = 3
1988-2003: EOD 10 - extent (1988-2003) = 30-33
2004+: CS extension (2004+) = 300-330
**Stage IV**
1983-1987: EOD 4 - extent (1983-1987) = 8
1988-2003: EOD 10 - extent (1988-2003) = 80
2004+: CS extension (2004+) = 800
**Unknown**
All other values

*Extent of Disease
**Clinical Stage

Supplementary Table 2 : Time-interval overall survival (OS) and cancer-specific survival (CSS) across all patient characteristics using the Kaplan-Meier (KM) curves: NLPHL=Nodular Lymphocytic Predominant Hodgkin Lymphoma, LR= Lymphocyte-rich, LD= Lymphocyte-depleted, MC= Mixed cellularity, NS= Nodular sclerosis, cHL= classical Hodgkin Lymphoma, **LN= Lymph Nodes, HFN= Head, face, and neck, IT= Intrathoracic, IAB= Intra-abdominal, AA= Axilla or arm, IL= Inguinal region or leg, P= Pelvic, Other*= Includes American Indian, AK Native, Asian, and Pacific Islander, Not married**= Includes single, widowed, separated, and divorced.**

Characteristic	*1-year OS*	*5-year OS*	*10-year OS*	*P-value*	*1-year CSS*	*5-year CSS*	*10-year CSS*	*P-value*
Patient age (years)				*< 0.0001*				*< 0.0001*
≤ 20	*0.9917*	*0.96*	*0.9373*		*0.9947*	*0.9677*	*0.9536*	
21-40	*0.9811*	*0.9243*	*0.8897*		*0.9873*	*0.9427*	*0.9236*	
41-60	*0.9399*	*0.8366*	*0.7311*		*0.9599*	*0.8911*	*0.8456*	
61-80	*0.7724*	*0.5511*	*0.3626*		*0.834*	*0.6839*	*0.6146*	
≥ 81	*0.5276*	*0.2215*	*0.045*		*0.6463*	*0.4533*	*0.278*	
Year of diagnosis				*< 0.0001*				*< 0.0001*
1983-1987	*0.9065*	*0.7809*	*0.7009*		*0.9297*	*0.8391*	*0.801*	
1988-2003	*0.93*	*0.8274*	*0.7532*		*0.9525*	*0.8842*	*0.8541*	
2004-2010	*0.9248*	*0.8501*	*N/A*		*0.9467*	*0.8974*	*N/A*	
Patient gender				*< 0.0001*				*0.4496*
Male	*0.9269*	*0.8206*	*0.7364*		*0.9511*	*0.8832*	*0.8467*	
Female	*0.9239*	*0.8377*	*0.7733*		*0.944*	*0.8814*	*0.8563*	
Race				*0.0029*				*0.0648*
White	*0.9248*	*0.8276*	*0.753*		*0.9474*	*0.8825*	*0.8519*	
Black	*0.9333*	*0.8276*	*0.7473*		*0.9524*	*0.877*	*0.8388*	
*Other**	*0.9083*	*0.8103*	*0.7236*		*0.9419*	*0.8778*	*0.8385*	
Unknown	*0.9897*	*0.9768*	*0.8966*		*0.9897*	*0.9768*	*0.9768*	
Lymphoma Subtype recoded				*< 0.0001*				*< 0.0001*
NLPHL	*0.9849*	*0.9425*	*0.8754*		*0.9945*	*0.9777*	*0.9534*	
LR	*0.9552*	*0.8526*	*0.7702*		*0.9776*	*0.9267*	*0.9081*	
MC	*0.8859*	*0.7505*	*0.6462*		*0.9206*	*0.8296*	*0.7925*	
LD	*0.6847*	*0.5863*	*0.4333*		*0.7249*	*0.6473*	*0.5484*	
NS	*0.9494*	*0.8631*	*0.8021*		*0.9655*	*0.9038*	*0.8768*	
cHL, NOS	*0.871*	*0.7586*	*0.6707*		*0.9047*	*0.8317*	*0.7831*	
Radiation therapy				*< 0.0001*				*< 0.0001*
Radiotherapy	*0.9726*	*0.8904*	*0.8197*		*0.9837*	*0.9331*	*0.907*	
No Radiotherapy	*0.8862*	*0.7746*	*0.6926*		*0.9177*	*0.8382*	*0.8007*	
Surgery				*< 0.0001*				*< 0.0001*
Surgery	*0.9775*	*0.8982*	*0.8304*		*0.9871*	*0.9389*	*0.9149*	
No Surgery	*0.9128*	*0.8106*	*0.7336*		*0.9383*	*0.8682*	*0.8351*	
Sequence number				*< 0.0001*				*0.0156*
One primary only	*0.9218*	*0.8271*	*0.7655*		*0.9447*	*0.8799*	*0.854*	
First of two or more primaries	*0.9629*	*0.8412*	*0.6758*		*0.9805*	*0.909*	*0.8391*	

Marital status at diagnosis				*0.008*				*0.475*
*Not married***	*0.9228*	*0.8236*	*0.7573*		*0.9433*	*0.8753*	*0.845*	
Married	*0.9288*	*0.8331*	*0.7477*		*0.9535*	*0.8905*	*0.8578*	
Stage				*< 0.0001*				*< 0.0001*
Unknown	*0.9135*	*0.7996*	*0.7243*		*0.9416*	*0.864*	*0.8298*	
I	*0.9452*	*0.8553*	*0.7799*		*0.9647*	*0.9087*	*0.8816*	
II	*0.9374*	*0.8523*	*0.7796*		*0.9574*	*0.899*	*0.8636*	
III	*0.8962*	*0.7669*	*0.6937*		*0.9276*	*0.8297*	*0.7977*	
IV	*0.7871*	*0.6352*	*0.548*		*0.8255*	*0.7018*	*0.6501*	
Primary LN site				*< 0.0001*				*< 0.0001*
LNs of IL	*0.8975*	*0.7477*	*0.6452*		*0.9293*	*0.8257*	*0.7723*	
LNs of HFN	*0.9517*	*0.8685*	*0.7945*		*0.9688*	*0.9173*	*0.8888*	
IT LNs	*0.9383*	*0.8522*	*0.7932*		*0.9559*	*0.8892*	*0.8595*	
IAB LNs	*0.7001*	*0.5589*	*0.4323*		*0.7532*	*0.6384*	*0.5769*	
LNs of AA	*0.8968*	*0.7621*	*0.6896*		*0.9307*	*0.8405*	*0.8157*	
P LNs	*0.7781*	*0.6695*	*0.5565*		*0.8114*	*0.7464*	*0.6932*	

Supplementary figure 2: Kaplan-Meier Curves for OS based on all included basic patient characteristics:

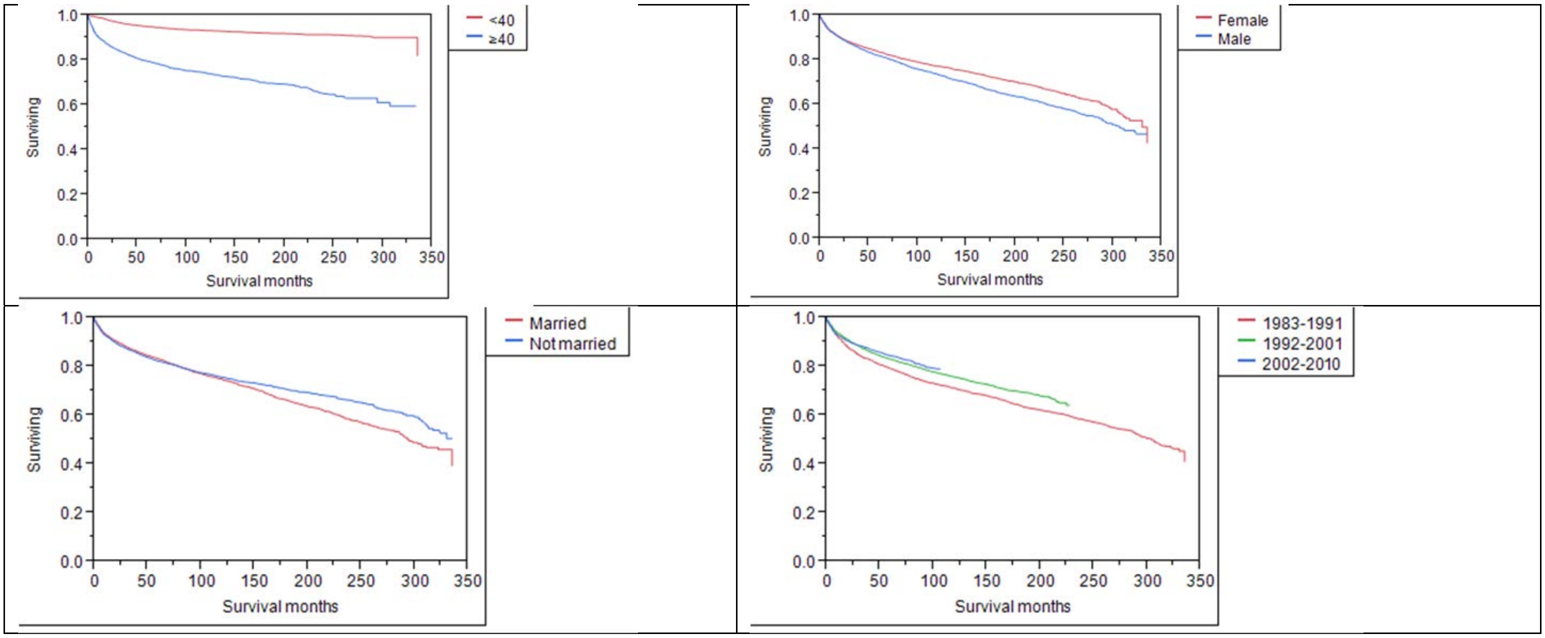

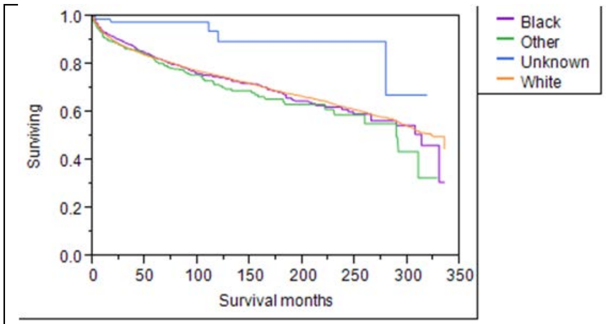

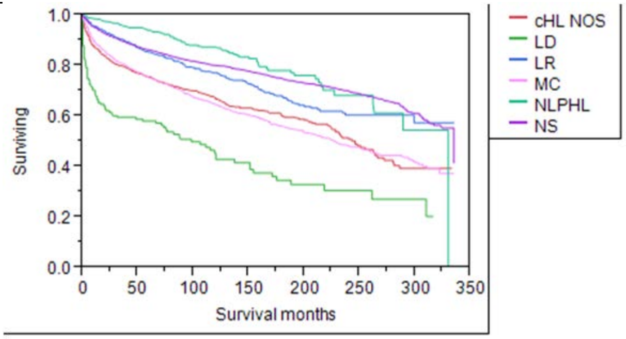

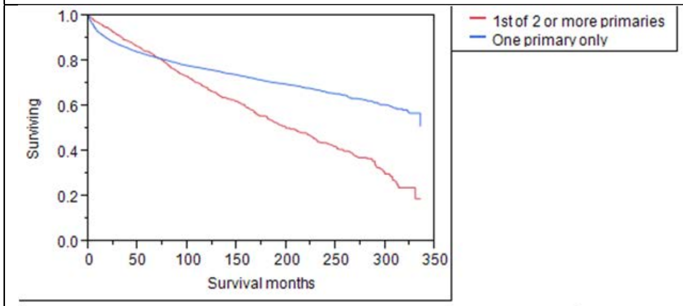

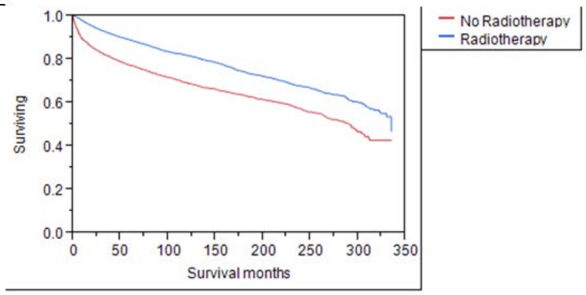

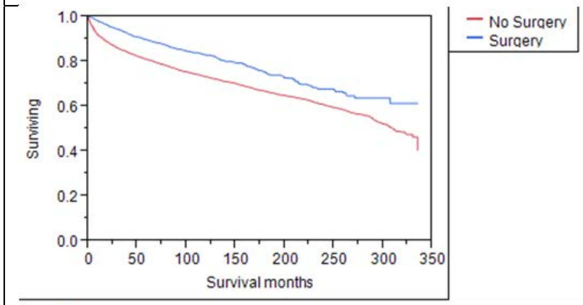

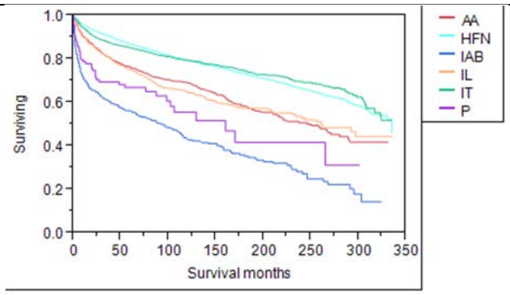

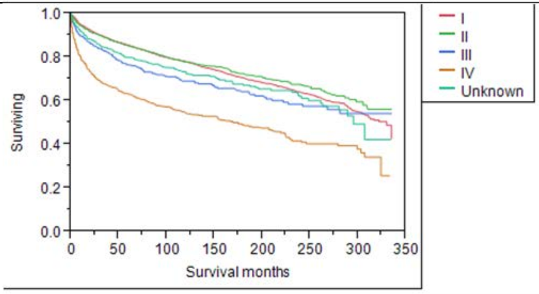

All Log Rank tests are statistically significant for different levels of the study cohort characteristics.

Figure 3: Kaplan-Meier Curves for CSS based on all included basic patient characteristics:

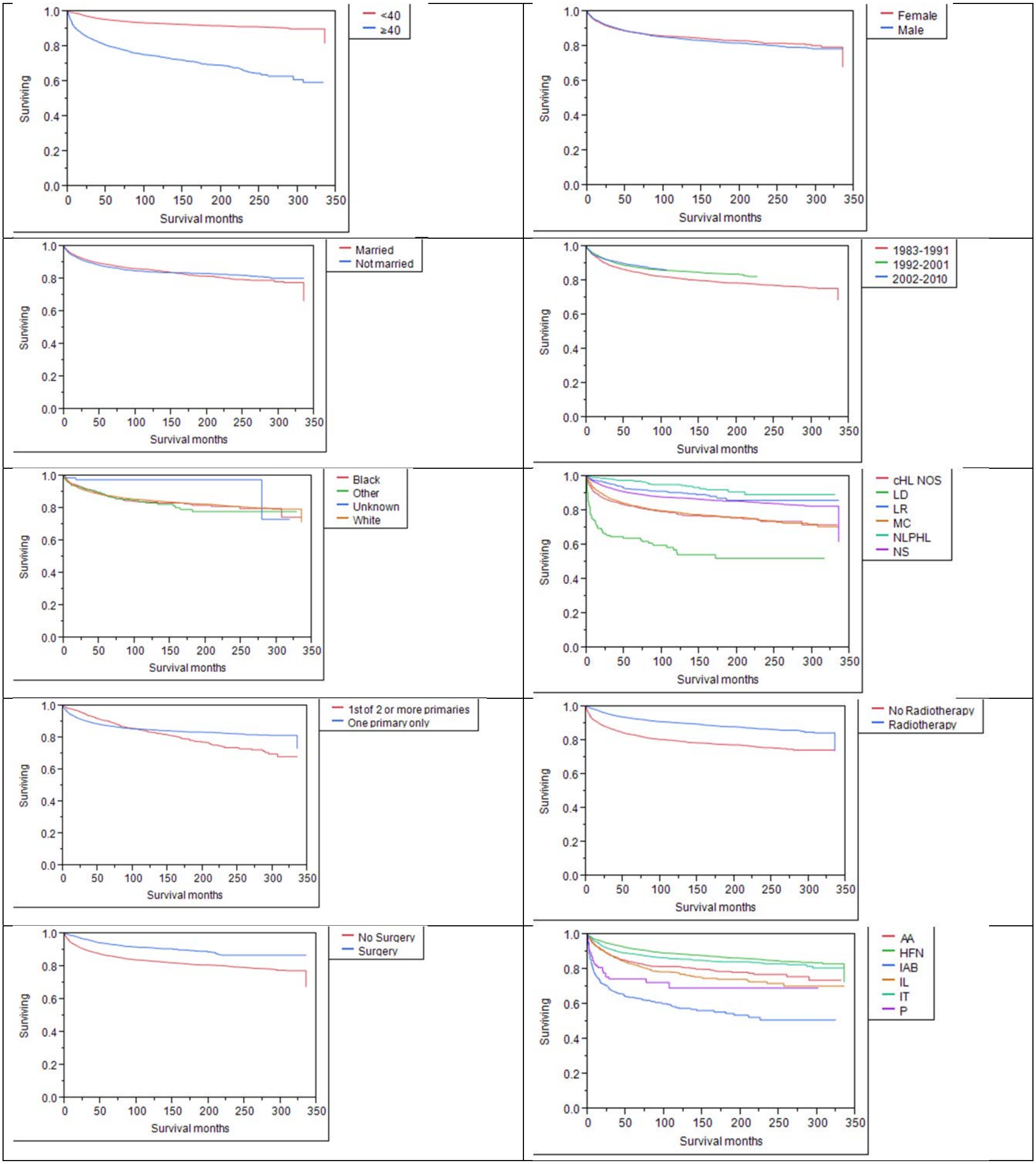

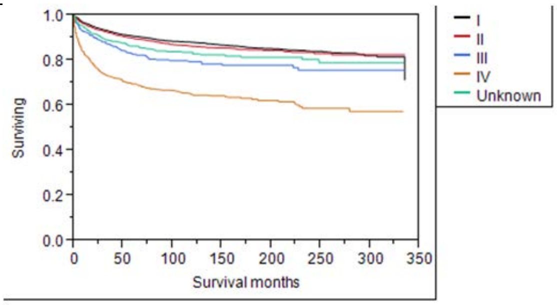		Log Rank tests resembling different levels of the study cohort characteristics were statistically significant with the exception of patients' gender, race, and marital status.
